# Interfacial activation of M37 lipase: A multi-scale simulation study

**DOI:** 10.1016/j.bbamem.2016.12.012

**Published:** 2017-03

**Authors:** Nathalie Willems, Mickaël Lelimousin, Heidi Koldsø, Mark S.P. Sansom

**Affiliations:** Department of Biochemistry, University of Oxford, Oxford, United Kingdom

**Keywords:** Molecular dynamics, Conformational changes, Interfacial interactions, Substrate binding, Enzyme activation

## Abstract

Lipases are enzymes of biotechnological importance that function at the interface formed between hydrophobic and aqueous environments. Hydrophobic interfaces can induce structural transitions in lipases that result in an increase in enzyme activity, although the detailed mechanism of this process is currently not well understood for many lipases. Here, we present a multi-scale molecular dynamics simulation study of how different interfaces affect the conformational dynamics of the psychrophilic lipase M37. Our simulations show that M37 lipase is able to interact both with anionic lipid bilayers and with triglyceride surfaces. Interfacial interactions with triglyceride surfaces promote large-scale motions of the lid region of M37, spanning residues 235–283, revealing an entry pathway to the catalytic site for substrates. Importantly, these results suggest a potential activation mechanism for M37 that deviates from other related enzymes, such as *Thermomyces lanuginosus* lipase. We also investigated substrate binding in M37 by using steered MD simulations, confirming the open state of this lipase. The exposure of hydrophobic residues within lid and active site flap regions (residues 94–110) during the activation process provides insights into the functional effect of hydrophobic surfaces on lipase activation.

## Highlights

• M37 lipase preferentially interacts with anionic phospholipid bilayers.
• M37-bilayer interactions do not result in activation of the lipase.
• M37 lipase interactions with triglyceride interfaces result in structural changes.
• An interfacial activation mechanism of M37 is proposed, allowing substrate access.

## Graphical abstract

**Figure:**
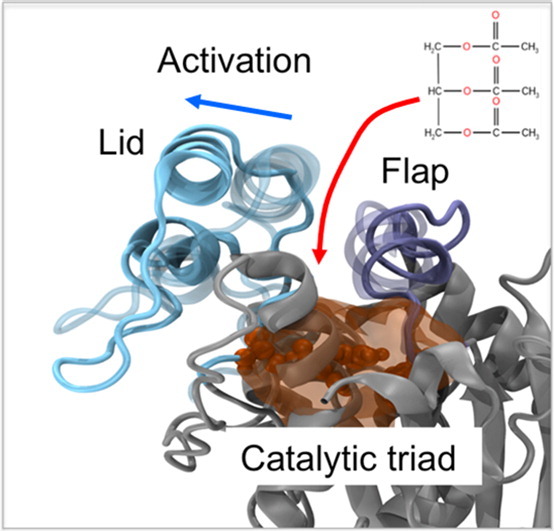

## 1 Introduction

Interfacial enzymes are water-soluble proteins that catalyse reactions at interfaces, such as lipid membranes [1], [2], [3]. Lipases are amongst the most studied interfacial enzymes, both due to their importance in fat metabolism and human disorders, such as Wolman's disease, and in biotechnological industry [1], [4]. Beyond their cellular roles, lipases can also catalyse a range of reactions acting on a range of unnatural substrates. This has resulted in the application of lipases in various industries, including the food, detergent, and pharmaceutical sectors [5], [6], [7]. However, the full potential of these enzymes has yet to be explored, in part because the molecular interactions that favour lipase activation at different interfaces are not completely understood [1], [8].

**Fig. 1:**
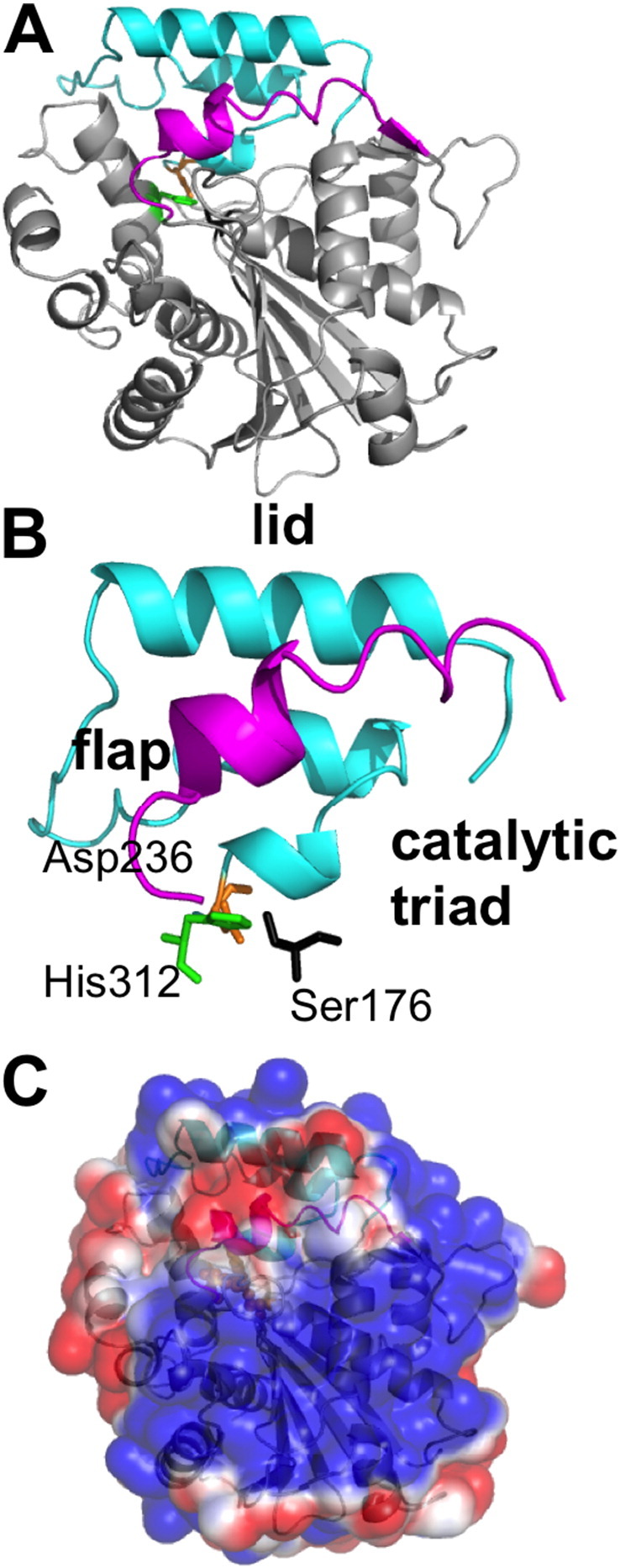
Crystal structure of M37 lipase. (A) Crystal structure of M37 lipase (PDB: 2ORY, 2.2 Å resolution) highlighting the lid (cyan) and active site flap (magenta) regions. (B) Close up of the lid (residues 235–283) and active site flap (residues 94–110) regions of M37 with the catalytic triad (Ser174, Asp236, and His312) residues (black, orange, and green sticks respectively). (C) Electrostatic surface of M37 (calculated using APBS [11]) shown in the same orientation as in A (blue: positive, red: negative, scaling from + 1 to − 1).

Among fifty or so bacterial lipases that have recently been identified, the M37 protein from *Photobacterium lipolyticum* represents an interesting example to explore interfacial activation. M37 exhibits a low activation energy towards triglyceride substrates, stability in non-aqueous solvents, and catalytic activity at low temperatures, due to its apparent ability to function at low temperatures (psychrophilicity) [9]. Some unique structural features of M37 are thought to underlie these properties [10]. As is commonly the case in lipase structures, the protein contains a so-called amphipathic lid region that covers the catalytic residues of the active site, in addition to a α-helical ‘flap’ region (Fig. 1).

It is thought that the lid and flap regions determine interfacial activation of M37 [10]. In general, interactions of lipases with hydrophobic interfaces are thought to result in a conformational transition in the lid region, exposing the active site and allowing substrate molecules to bind. However, the exact nature and order of these steps is thought to differ for different lipases and thus a general mechanism of interfacial activation remains elusive. Moreover, the variation in the interfacial interactions with natural and unnatural substrates remains to be determined. In this context, molecular dynamics (MD) simulations provide a valuable approach to probe the underlying dynamic processes determining the activity of enzymes [12], [13], and their behaviour in different environments. Simulations have been used to identify solvent-induced effects on interfacial activation for lipases closely related to M37 (e.g. *Thermomyces lanuginosus* lipase (TLL)) [14]. Computational approaches have also provided important insight on interfacial interactions, which contributed to unveiling functionally important motions in lipases [14], [15], [16], [17]. Therefore, the analysis of M37 dynamics over different simulation timescales might be expected to reveal key aspects of its activation mechanism.

Here, we used a multi-scale simulation framework to investigate the dynamics of M37 in different environments, including at different interfaces and in aqueous solution. First, the interfacial behaviour of the lipase was analysed with two different phospholipid bilayers. We observed that M37 preferentially interacts with negatively charged membranes, correlating with experimental studies of a related fungal lipase [18], [19], [20]. Second, interfacial interactions with a natural substrate (tributyrin) revealed functionally relevant motions of the enzyme. A large-scale motion of the lid region was identified to open the initially closed conformation of M37, uncovering the entry pathway of the catalytic site to the natural substrate. This novel mechanism of activation provides new insights that which will enable rational engineering of the psychrophilic M37 lipase.

## 2 Computational methods

### 2.1 Parameterisation of CG models

The crystal structure of M37 was downloaded from the Protein Data Bank (PDB: 2ORY) and converted to atomistic (AT) and coarse-grained (CG) representations. The Martinize.py script was used to convert the M37 crystal structure to CG representation (available at: http://md.chem.rug.nl/index.php/tools2/proteins-and-bilayers). A molecular dynamics (MD) simulation of M37 in aqueous solution was initially performed at AT level (see AT-MD parameters below). The Martini 2.2 forcefield [21], [22] was used for the CG simulations, which employs an elastic network model to maintain the secondary and tertiary structures of proteins. This was parameterised for M37 using the ElNeDyn framework (ENM) [23]. Different cut-off radii and force constants were tested in CG simulations, using the AT simulation as a reference for protein dynamics (Fig. S1). The root mean square fluctuations (RMSF) and root mean square deviations (RMSD) of Cα atoms of M37 were calculated at both AT and CG levels and then compared. We found that the best ENM parameters to use in the CG simulations were a cut-off radius of 0.95 nm and a force constant of 550 kJ mol^− 1^nm^− 2^.

### 2.2 CG-MD simulations

The Martini forcefield maps, on average, four non-hydrogen atoms to a single particle, and was employed using standard parameters for bonding and non-bonding interactions. Self-assembly simulations were performed to construct two solvated bilayers containing 512 lipids each. A purely zwitterionic bilayer (100% DOPC) and an anionic bilayer (20% DOPG: 80% DOPC) were built to investigate the effect of surface charge on M37 binding. Both bilayers were symmetric in terms of lipid composition of the upper and lower leaflets, resulting in a distribution of 257 lipids (191 PC + 66 PG) in the upper leaflet and 255 lipids (193 PC + 62 PG) in the lower leaflet (measured after 1 μs simulation time). The enzyme was initially placed 3 nm above the preformed bilayers and then the system was solvated. Counter ions were added to neutralise the charge of the systems. Two different starting orientations of M37 were simulated to minimise any bias occurring from the initial position. Energy minimisation was performed for 200 steps followed by equilibration for 10,000 steps. Ten replicas of each starting orientation were simulated in the NpT ensemble at 310 K and 1 bar, resulting in a set of 20 CG-MD simulations of 5 μs each. A leapfrog algorithm was used to integrate Newton's equations of motion, with a time step of 20 fs. The Berendsen thermostat [24] was used for temperature coupling with a weak coupling constant of 1.0 ps. Semi-isotropic pressure coupling was applied using the Berendsen barostat with a 1.0 ps coupling constant and a compressibility of 3 × 10^− 4^ bar.

### 2.3 AT-MD simulations

The last frames of selected M37-anionic bilayer CG simulations were converted to AT representations using a fragment-based approach [25]. The GROMOS 53A6 forcefield and SPC water model were used for all simulations (see Table 1) [26], [27], [28]. Non-bonding interactions were considered through a buffered Verlet scheme [29]. Long-range electrostatic interactions were treated using the particle mesh Ewald method [30] with a real-space cut-off of 1 nm, a Fourier spacing of 0.12 nm and a fourth-order spline interpolation. Short-range van der Waals interactions were treated within a cut-off range of 1 nm. The V-rescale thermostat [31] was used for temperature coupling and the Parrinello-Rahman barostat [32] for semi-isotropic (lipid simulations) or isotropic (tributyrin simulations) pressure coupling. The LINCS algorithm [33] was used to constrain bond lengths. A leapfrog algorithm was used to integrate Newton's equations of motion, with a time step of 2 fs. All simulations were equilibrated for 1 ns during which all the atoms in the protein were position restrained using a force constant of 1000 kJ mol^− 1^nm^− 2^, prior to performing 200 ns of unrestrained simulations. Previous studies have indicated that M37 has an optimum pH of 9–10 at 303 K (30 °C), but remained stable within a broad pH range of 2–11 [9], [34]. All simulations reported in this study, including the CG simulations, were performed at pH 7. The same pH has been used in previous computational studies of related lipases at tributyrin interfaces [35], [36], [37].

### 2.4 Steered AT-MD simulations

Steered MD (SMD) simulations were performed using the Plumed 2.1 plugin for GROMACS [38], [39]. The equilibrated structure of the M37 simulations in water was used as the starting point, applying identical simulation parameters as for the AT-MD simulations mentioned above. All SMD simulations were performed using the GROMOS 54A7 forcefield [40], [41]. Different collective variables (CVs) were tested to probe the lid and flap motions occurring though opening of the closed form of M37 (crystal structure). A distance CV was defined between the COM of the lid helix closest to the active site flap (residues 264–279) and the helix of the active site flap itself (residues 94–110; Fig. 1). A harmonic bias potential was applied to the CVs, moving at a constant velocity of 1 nm ns^− 1^ with a spring stiffness ranging from 500 to 1000 kJ mol^− 1^nm^− 2^ (Table 1). Additionally, a pseudo-dihedral CV was tested, defined by specified Cα positions of residues within the hinge regions of the active site flap (Fig. S7). The limit values of both the distance and the pseudo-dihedral CVs were estimated by producing an opened M37 model based on structural alignment of the active site flap region with an open form of TLL [42] using the sculpting tool in PyMOL (www.pymol.org).

### 2.5 Tributyrin setup

The tributyrin GROMOS 54A7 AT forcefield topology and coordinate files using parameters were downloaded from the Automated Topology Builder website (http://compbio.biosci.uq.edu.au/atb/) [43], [44], [45]. A tributyrin layer was formed by randomly inserting 731 tributyrin molecules into a 10x10x5 nm cubic box. After steepest descent energy minimisation, 1 ns of NvT equilibration at 298 K was performed, after which the z-dimension of the box was extended to 10 nm and the system solvated with SPC water. An additional 1 ns of NpT simulation ensured that the density of tributyrin equilibrated to 1026 g L^− 1^ (experimental value at 298 K = 1027 g L^− 1^ [46]). M37 was then positioned 1 nm above the tributyrin layer in the centre of the box (distance measured from the bottom of the protein to the top of the layer), before performing 200 ns of MD simulation.

### 2.6 Docking calculations

All docking studies were performed using GOLD [47], [48]. Protein models consisted of either the last frame of the M37-tributyrin simulations or an equilibrated structure of the closed form. The protein models were prepared by adding hydrogens with the default settings of the GOLD program. Docking of one tributyrin molecule in the active site cavity, defined by 1 nm radius from the position of residue Ile235, was performed using the CHEMPLP scoring function [49] (Fig. S11).

## 3 Results

### 3.1 Interfacial interactions of M37 with lipid bilayers explored via coarse-grained simulations

We used coarse-grained (CG)-MD simulations to investigate the diffusional encounter of the M37 lipase with pre-formed lipid bilayers. Both zwitterionic (100% PC) and anionic (20% PG: 80% PC) lipid bilayers were tested to explore the effect of surface charge on the enzyme binding and interfacial interactions (see Table 1).

Only transient interactions occurred with the zwitterionic bilayers were observed (Fig. S2). In contrast, we observed preferential and long-lasting interactions of the protein with the anionic bilayer. Therefore the following analysis refers to simulations with anionic (PC/PG) bilayers only. Binding events occurred over the course of all the 20 replicate CG-MD simulations, within a time ranging from 100 ns to 4 μs (Fig. S3). Once bound the protein did not dissociate from the anionic lipid bilayer.

**Fig. 2:**
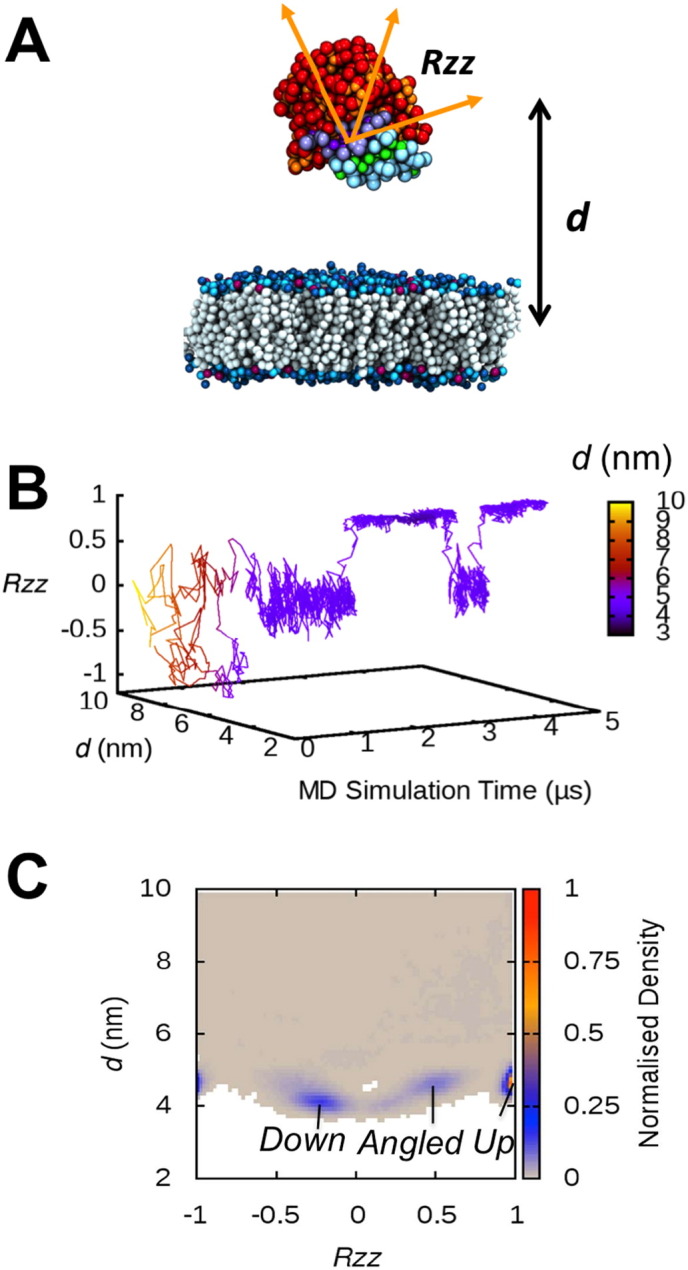
Binding analysis of CG-MD simulations of M37 with anionic lipid bilayers. (A) Initial system configuration for the CG-MD simulations of M37 binding to lipid bilayers. The figure schematically represents the CVs (*d* and *Rzz*) used to describe the motion and orientation of M37. The metric *d* was calculated as the z-component of the distance between the COM of the protein relative to the COM of the bilayer. A rotation matrix was calculated as a function of the *Rzz* angle that defines the transition from a given orientation of the enzyme (orange arrows) to a reference orientation. When *Rzz* = 1, the enzyme adopts the orientation of the reference structure. (B) Time evolution of *d* and *Rzz* for the protein during one of the CG-MD simulations (raw data shown in Fig. S4). The protein switches from the *Up* to the *Angled* binding orientation during the simulation, reflected in the value of *Rzz*. (C) The normalised density of each binding orientation calculated as a function of *d* and *Rzz* derived from the ensemble of the 20 CG-MD simulations. The lipid headgroups of the upper leaflet of the lipid bilayer correspond to the bottom of the map (*d* ≈ 2 nm).

In order to analyse the binding mechanisms and the interfacial orientations of M37 on the anionic membrane surface in more detail, two collective variables (CV) were defined: a translational CV (*d*), defined as the z-component of the distance between the centres of mass (COM) of the protein and of the bilayer; and a rotational CV (*Rzz*), obtained from the rotation matrix calculated with respect to a reference orientation of the protein (Fig. 2A). Before binding to the lipid bilayer, the protein underwent translational and rotational motions in solution as anticipated, assessed by variations in *d* and *Rzz*. In contrast, upon binding to the anionic membrane, the lipase showed a small number of canonical binding orientations, although conversions between these configurations were observed (Fig. 2B).

**Fig. 3:**
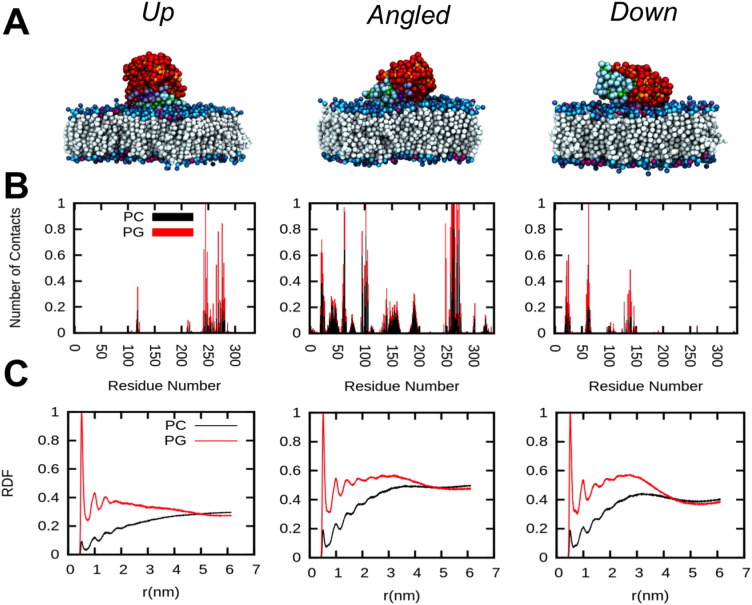
Interaction analysis of CG-MD simulations of M37 with anionic lipid bilayers. (A) Representative structures of the three different orientations of M37, observed in the CG-MD simulations, when bound to the anionic lipid bilayer (20% PG: 80% PC). (B) Normalised number of contacts between the residues of M37 and either PC or PG lipids, as a function of the protein residue number. (C) Normalised radial distribution functions of lipid density as a function of the distance of the protein to either the PC or the PG headgroups (choline and glycerol, respectively). All frames corresponding to one binding orientation were combined to calculate the statistics for a given population.

The ensemble of 20 CG-MD simulations allowed us to calculate a 2-dimensional landscape representing the normalised density of the binding orientations, as a function of *d* and *Rzz* (Fig. 2C). Together with a visual inspection of the simulations, this analysis revealed three major binding configurations of the lipase on the bilayer surface, which we have termed *Up*, *Angled*, and *Down* (Figs. 2C and 3A). The density map indicates that the *Up* orientation is the most frequently sampled orientation within the simulation ensemble.

The three identified orientations were further characterised by cluster analyses, i.e. extracting and concatenating all trajectory frames corresponding to a specific orientation from the whole simulation ensemble. First, the average number of contacts between the protein and the lipid headgroups were calculated for each orientation (Fig. 3B). Second, average radial distribution functions (RDF) were computed considering the two lipid types (PC and PG) individually (Fig. 3C). Contact analysis indicated that hydrophobic and basic residues mediate interactions with PG lipids, while mainly hydrophobic amino acids formed contacts with PC molecules (Fig. 3B). The *Up* and *Down* binding orientations seemed to involve a larger number of basic residues mediating interactions with anionic PG lipids relative to the *Angled* orientation (Fig. S5). Additionally, RDF calculations indicated a higher density of PG lipids in the vicinity of the protein, relative to PC lipids, for all three of the binding orientations (Fig. 3C). Overall these data suggests that electrostatic interactions promote interfacial interactions, as has been suggested experimentally for other related lipases [18], [19], [50].

### 3.2 Atomistic simulations of the conformational dynamics of bound M37

**Fig. 4:**
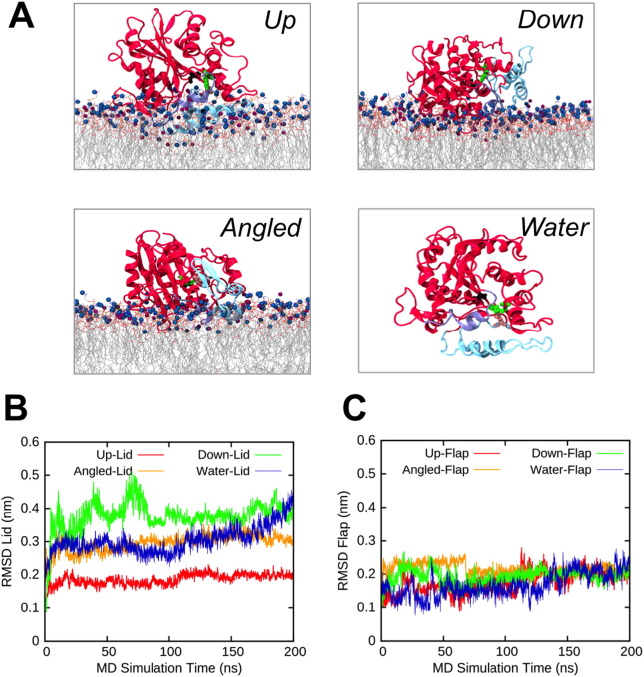
Structural analysis of AT-MD simulations of M37 with anionic lipid bilayers. (A) Images showing the final frame of AT-MD simulations of M37 with anionic bilayers (after 200 ns), representing the *Up*, *Angled*, and *Down* binding orientation, and the final frame of an AT simulation of M37 in water. The lid region of M37 is coloured in cyan and the active site flap region in purple; the rest of the protein is coloured in red (cartoon representations). The lipid tails are shown as sticks; PC headgroups are shown as points coloured in blue, and PG headgroups are coloured in purple. Below, time evolution of the RMSD of Cα atoms within the lid (**B**) and active site flap (**C**) regions.

Atomistic (AT) simulations were initiated from representative CG simulation frames for each binding orientation, with the aim of studying the interfacial interactions in more detail [25]. The atomistic simulations showed a general agreement with the CG simulations regarding the protein-membrane interactions (Fig. S5), for each of the three binding orientations (Fig. 4A).

The atomistic simulations (200 ns) also allowed us to analyse the conformational dynamics of M37 in the three binding orientations, and compare them to simulations of the protein in water. Interestingly, all binding orientations, except for the *Up* orientation, exhibited larger structural fluctuations in the lid region compared to the active site flap region, as reflected by RMSD calculations of these regions (Fig. 4B and C). More specifically, we observed that increased interactions with the membrane lipids for the protein in *Up* orientation, and to a lesser extent in *Angled* orientation, resulted in suppressed motions of the lid region, as compared to the *Down* orientation (Fig. 4A and B). In the latter orientation, the solvent exposure of the lid region induces fluctuations similar to those observed for the protein in solution.

Overall these results suggest that interfacial interactions can affect the dynamics of the lid but *not* of the active site flap region. By inspecting the activation mechanisms of related enzymes (e.g. TLL or *Rhizomucor miehei* lipase (RML)) [10], one might rather expect functionally important motions to occur in the active site flap. The increase in RMSD of the lid region for the protein in the *Down* orientation and in water is attributed to partial unfolding of the lid region during these relatively long simulations, as is shown in Fig. S6. Alignment of the final structures from the end of the atomistic bilayer simulations with the crystal structure of M37 indicates that neither the active site flap region nor the lid region were significantly displaced by any of the interfacial interactions (Fig. S6). Thus, in order to identify functional motions of M37, we investigated longer timescale motions of the lid and active site flap regions.

### 3.3 Functionally relevant motions of M37 in water

Steered molecular dynamics (SMD) simulations may be employed to investigate slow and low frequency motions of biomolecules. A steered MD simulation accelerates biomolecular motions by applying an external force along a collective variable (CV) of interest, driving biologically relevant motions within shorter simulation times [51].

**Fig. 5:**
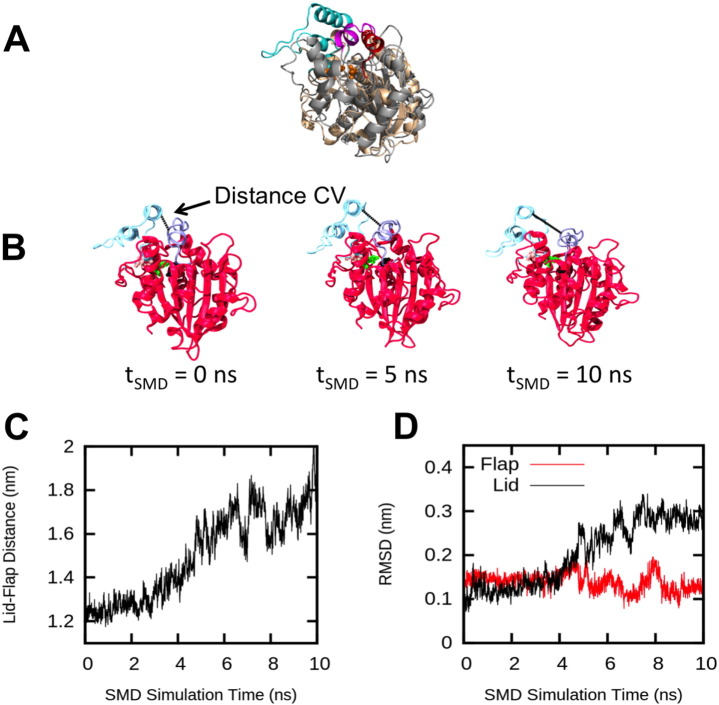
Steered MD simulations of M37 in water. (A) Structural alignment of the closed M37 crystal structure (PDB: 2ORY; grey) with the open TLL crystal structure (PDB: 1DT5; light orange) was done using the DALI server (http://ekhidna.biocenter.helsinki.fi/dali_server/) [52], [53]. The M37 lid and active site flap regions are coloured in cyan and magenta, respectively, and the TLL active site flap region is shown in red. (B) Intermediate structures of M37 in water (omitted for clarity) from a selected unrestrained SMD simulation using the lid-flap distance as a CV (schematically represented by a dotted line). (C) Distance CV calculated between the COM of the lid and the active site flap helices, as a function of the simulation time. (D) Time evolution of the RMSD of the lid and flap regions.

We wished to define CVs able to model possible long timescale motions of the lid and the active site flap regions, which could lead to opening of the closed form (crystal structure) of M37 in water. Since there are no crystal structures of the open form of M37, the closed form was compared to the open conformation of the structurally similar *Thermomyces* *lanuginosus* lipase (TLL; PDB: 1DT5) [42]. Alignment and comparison of the two structures (Fig. 5A) revealed that the lid region identified in M37 is not present in TLL. However, the active site flap region within M37 could be aligned to the lid region of TLL, showing an evident potential shift in its position. We used this information as a starting point to define suitable CVs for the SMD simulations.

We defined a distance CV in order to model the opening motion of M37 as the distance between the COM of the helical parts of the lid (residues 262–278) and of the active site flap (residues 94–110). The SMD protocol enabled a gradual extension of the distance between lid and flap helices (Fig. 5B–C). We performed several SMD simulations using this distance CV, which all showed very similar trends (5 replicates). In agreement with previous results, the active site flap did not significantly move from its original position. Instead, the lid region showed a large displacement, maintaining its α-helical fold during the simulation (Fig. 5D). We observed that the initial contacts between lid and flap mainly involved polar and hydrophobic residues and these contacts broke as the helices moved away from each other (Figs. S9 and S10). Indeed, the final exposure of hydrophobic residues to the solvent suggests that interactions with hydrophobic interfaces are necessary to bring about lipase activation. Overall this indicates that the displacement of the lid region is a likely structural determinant of M37 activation.

### 3.4 Interfacial activation of M37 with a natural substrate

Beyond the intrinsic dynamics of M37 observed both in solution and at the interface with lipid bilayers, it is important to characterise the motions of the protein in contact with an interface formed by its natural substrate. In particular it has been reported that the related TLL enzyme could maintain an open active state at a triglyceride interface [35]. Therefore, we performed atomistic simulations with an interface composed tributyrin molecules, a triglyceride substrate of M37.

**Fig. 6:**
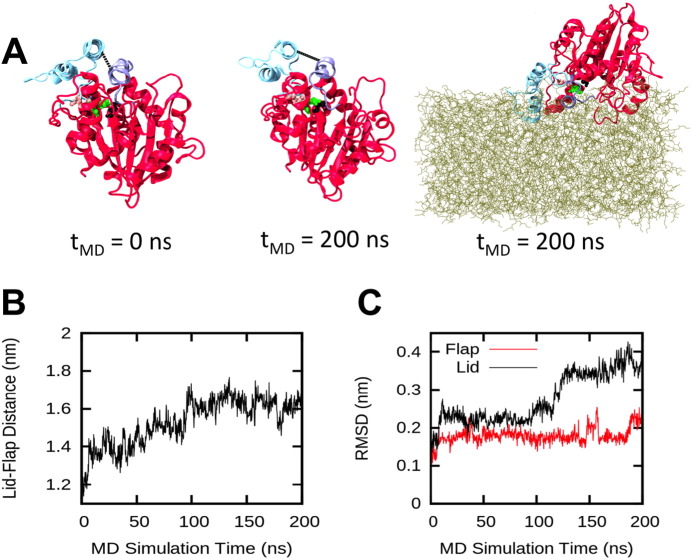
AT-MD simulations of M37 with a tributyrin interface. (A) Images showing the structure of M37 in the first and final frames of an atomistic M37-tributyrin simulation. Position of the lid (cyan) and active site flap (purple) regions are shown at 0 ns (left) and 200 ns (middle); the water and tributyrin molecules are omitted for clarity. Right: Snapshot of the final frame of an AT-MD simulation of M37 interactions with a tributyrin interface, showing the tributyrin layer (brown sticks) and the binding orientation of M37 at 200 ns. The lipase is coloured in red (cartoon representation), the active site flap in purple, and the lid region in cyan. (B) Time evolution of the distance CV calculated between the COM of the lid and the active site flap helices. (C) Time evolution of the RMSD of Cα atoms in the lid and flap regions.

The lipase was initially positioned above the layer of tributyrin. Within the first 20 ns of the AT-MD simulations M37 associated with the tributyrin layer, which induced a large-scale conformational change in the lid region of the lipase (Fig. 6A). The evolution of the distance between the COM of the lid region and of the active site flap was similar to that observed in the SMD simulations of M37 in water (Fig. 5C). Opening of the lipase structure occurred within the first 100 ns of simulation time, and was maintained for the duration of the simulation. This indicates that interfacial interactions with the tributyrin layer resulted in stabilisation of M37 in an open state. Consistent with the previously identified structural motions of the lipase, the conformational change corresponded to a displacement of the lid region, while the active site flap remained mainly static (Fig. 6C). These (unbiased) simulations with a natural substrate confirm the potential mobility of the lid region and its likely involvement in the activation mechanism of M37.

The binding orientation of M37 on the tributyrin surface was similar to the *Angled* orientation identified on an anionic lipid bilayer (Fig. 4). However, the large-scale displacement of the lid region captured on the tributyrin layer was not observed for the related orientation on the anionic bilayer (Fig. S6). These results show that the nature of the interface is essential for triggering functional motions of the bound lipase.

Docking calculations were also performed to predict binding poses of tributyrin substrate with both the closed form of M37 (crystal structure) and the open form observed at the end of the tributyrin layer simulations. Whereas several binding modes were identified in the active site of the open form (Fig. S11), the calculations failed to find any viable docking poses for the closed state. This indicates that the open form of M37, in which the lid is displaced, likely corresponds to an active state of the enzyme. However, in order to determine whether the structures bound to the tributyrin layer represent a partially or fully open state, we performed additional SMD simulations to investigate possible entry pathways of the tributyrin substrate molecule into the active site. A similar protocol has been previously applied to study activation pathways and substrate binding mechanisms of other lipases [54], [55]. Here, we defined M37 as strictly *opened* if the lid region did not impede substrate entry into the active site.

**Fig. 7:**
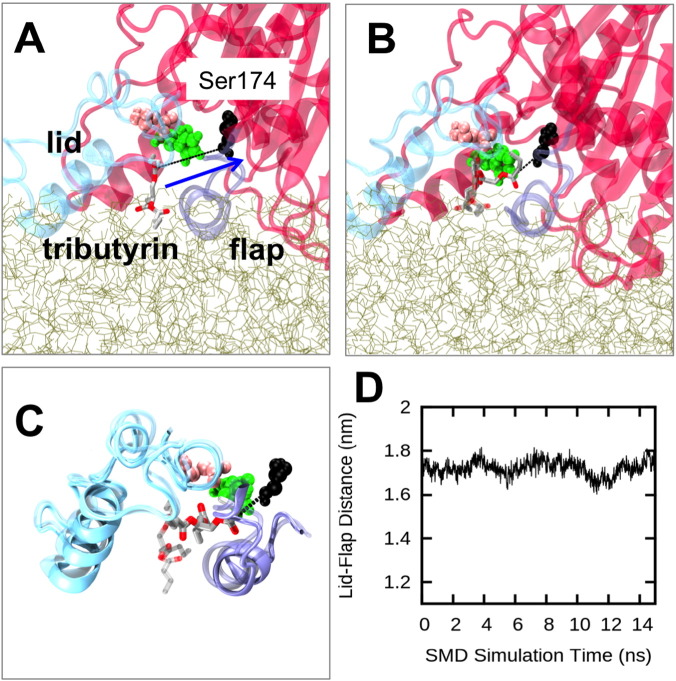
Substrate entry into catalytic site of interfacially bound M37. (A) Setup of the SMD simulations in which a tributyrin molecule (grey and red sticks) was pulled from the tributyrin layer (light brown sticks) towards the active site Ser174 residue. The catalytic residues Ser174 (black), Asp236 (pink), and His312 (green) are shown as van der Waals spheres. The arrow represents the direction of the pulling force and a dashed line indicates the distance between the tributyrin molecule and Ser174. (B) Starting (transparent sticks) and end positions (opaque sticks) of the pulled tributyrin molecule from a selected SMD simulation. (C) Cartoon representations of the starting (transparent) and end positions (opaque) of the lid (cyan), the active site flap (purple), and the tributyrin molecule (sticks) from the selected SMD simulation. (D) Distance CV calculated between the COM of the lid and the active site flap, as a function of the SMD simulation time.

We pulled a tributyrin molecule from the layer to which the enzyme was bound. This tributyrin molecule was initially wedged between the active site flap and the lid region at the end of the AT simulation, and moved during the SMD into the binding pocket, specifically towards the catalytic Ser174 residue (Fig. 7A). The SMD simulations showed complete entry of the substrate into the binding pocket (Fig. 7B). During this process both the lid and the active site flap regions moved only minimally (Fig. 7C). Consequently, the distance between the lid and flap regions showed only small variations (Fig. 7D). Therefore the M37 conformation observed at the end of the unbiased tributyrin simulations corresponds to a fully *open* form, able to bind substrate, which thus enables catalytic activity.

## 4 Discussion

Interactions of lipases with a range of different interfaces including lipid bilayers have been investigated using many experimental and computational techniques [20], [50], [56], [57], [58], [59]. However, the mechanisms of their association with lipid bilayer and related interfaces remain incompletely understood, especially in the context of lipase activation, although electrostatic interactions are generally considered important. This is exemplified by studies reporting that the TL lipase interacts with both POPG and POPC lipid vesicles, but is only active in the presence of POPG vesicles [18], [19]. This correlates nicely with our CG simulations, which showed that the related M37 enzyme could extensively interact with anionic PG-containing bilayers, but not with zwitterionic PC bilayers.

Studies of TLL using fluorescence microscopy suggested that this lipase could adopt different orientations on the lipid vesicles [18], comparable to the different binding orientations exhibited in our simulations of M37 on anionic lipid bilayers (i.e. *Up*, *Angled*, *Down*). Other computational studies have also revealed that lipases may exhibit varying orientations at different interfaces, which have different functional consequences regarding lipase activation [36], [60]. The amino acids mediating interfacial interactions were found to be different when comparing the interactions with PC vs. PG molecules within the anionic bilayer. Hydrophobic residues mainly showed interactions with PC molecules, whereas basic residues mediated interactions with the PG molecules, particularly for the *Angled* and *Up* states (Fig. S5). Therefore, the balance of hydrophobic and predominantly electrostatic interactions at the interface determined, for the most part, the orientation adopted by the enzyme (Fig. 3). The agreement with atomistic simulations suggests that the Martini model is able to correctly distinguish the various types of lipid-enzyme interactions at bilayer interfaces [61], [62]. It would also be of interest in future studies to investigate the effects of e.g. membrane curvature, which have been previously identified to affect lipase activity [18].

**Fig. 8:**
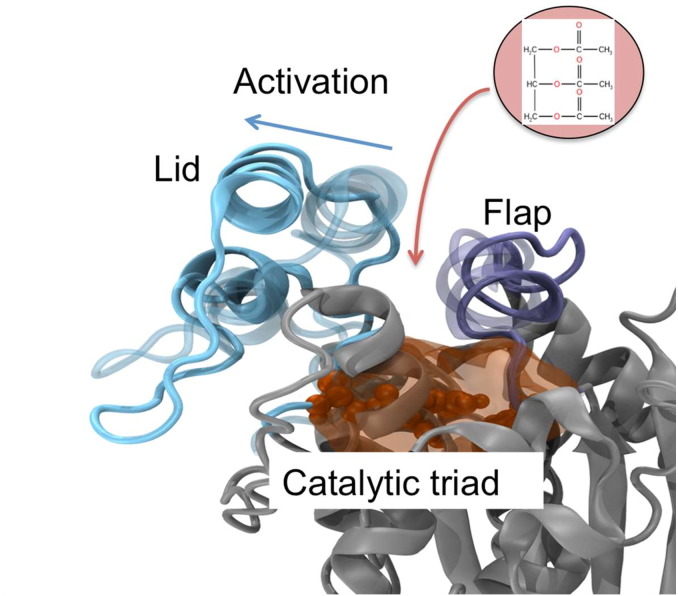
Proposed activation mechanism of M37 induced by lid displacement. The blue arrow shows the direction of the motion of the lid (cyan) that is required to uncover the underlying catalytic site (orange) for subsequent entry of substrates (indicated schematically by the red arrow and disc. The chemical structure of a triglyceride (triacetin) molecule is shown). The catalytic residues are shown in orange van der Waals spheres, and the binding pocket as an orange surface. The active site flap is coloured in purple. The initial positions of the lid and active site flap are shown as transparent, as identified from the closed crystal structure.

We were able to identify a possible activation pathway of M37 induced by lid displacement, which can be promoted by interfacial interactions with a tributyrin layer, a natural substrate for M37 (Fig. 5, Fig. 6). The lipase adopted a similar *Angled* orientation that was identified as a binding orientation in simulations with anionic phospholipid bilayers. This suggests a selection process in the interfacial binding orientations for the enzyme to become activated when a natural substrate interface is present. During activation, the lid motion is required to uncover the underlying catalytic site for subsequent entry of substrates (Fig. 7, Fig. 8). This conformational change is somewhat unexpected as activation of structurally similar enzymes e.g. TLL and *Rhizomucor miehei* lipase (RML) instead involves a motion of lid regions that align to the active site flap of M37 [42], [63], [64]. However, it is reasonable to suggest that M37 may display a novel activation pathway, given that the lid region of M37 (residues 235–283) is not found in these related enzymes. Furthermore, the active site flap also exhibited less flexibility relative to the lid region in the bilayer surface simulations, highlighting the possible functionality of the lid region, and the rigidity of the active site flap region. In addition, the hydrophobic residues that mediated contacts between the lid and the active site flap regions in the closed form of M37 became exposed to the surrounding solvent during the opening motion, induced by the lid region displacement (Fig. S10). Consequently, hydrophobic interfaces can play a decisive role in inducing the active form of such lipase, thus enhancing its activity.

The nature of the interface is clearly important, given that the lid opening motion was only seen at the substrate interface (tributyrin) but not at the phospholipid bilayer interface (Fig. S6). Similarly, soluble phospholipase A2 enzymes are allosterically modulated to adopt an “active” state by specific interfacial interactions with their natural substrates [65]. Our results suggest that the nature of the interactions with the anionic phospholipid bilayer suppresses lid motion, particularly in the *Angled* and *Up* orientations (Fig. 4B). Given that M37 is thought to be an extracellular lipase [9], the enzyme may predominantly interact with interfaces composed of its natural substrate in vivo, e.g. emulsions formed from short-chain triglycerides, or heterogeneous interfaces containing significant concentrations of triglyceride substrate. Our simulations thus correlate with the expectation that M37 exhibits functional motions with an interface formed of natural substrate, which are apparently not induced by interactions with a phospholipid interface. It is possible that the observation of different interfacial orientations of M37 with the anionic phospholipid bilayer is a result of the lack of interactions necessary to bring about displacement of the lid region. Contrastingly, only one binding orientation of M37 was seen with the tributyrin interface, likely stabilised by specific interactions that induced lid displacement. As such, although lipase adsorption can be observed for different interfaces, the functional consequences of these interactions can differ. Continued investigation of lipase interactions with biologically representative interfaces would be of interest to provide further insight into how the interface may affect the function of bacterial lipases in vivo.

To conclude, a multi-scale simulation approach allowed us to characterise interfacial interactions of M37 with different surfaces. The mechanism of association with anionic phospholipid membranes was modelled, revealing a distribution of binding orientations that determined the magnitude of local fluctuations observed in the lid region of M37. Interfacial interactions with a tributyrin layer resulted in large-scale motions of the lid, which opened the closed conformation of M37 and uncovered the entry pathway of the catalytic site to this natural substrate. The exposure of hydrophobic residues in the identified open state rationalises the functional effect of hydrophobic surfaces. It would be of great interest to characterise the free energy surface underlying M37 activation, particularly for the interfacially bound form. The SMD simulations performed in this study provide an initial description of the energetics underlying lid motion. However, these simulations were limited to the unbound form of M37 in water. This is because, upon interaction with the tributyrin interface, the lid region of M37 was observed to undergo almost immediate displacement, preventing comparative SMD simulations, which were initiated from the closed form of the lipase. Interestingly, initial characterisation of the free energy of lipase activation via potential of mean force calculations show that the underlying energy surface is quite complex, and may require refinement of the distance CV used in the SMD simulations. Indeed a recent study [66] has suggested that even for relatively simple processes involving protein/lipid, considerable attention to identification of suitable CVs is required if converged PMFs are to be obtained. Future developments in such approaches would also facilitate comparisons to lipase stability at other interfaces, and in solution. Overall however, the initial results represent a possible activation mechanism of a lipase that can be promoted by hydrophobic interfaces. This provides valuable insights into a relatively understudied, but industrially relevant psychrophilic lipase.

## Funding

This research was supported by the Biotechnology and Biological Sciences Research Council (BBSRC) [grant number BB/J014427/1].

## Transparency document

Transparency document. Image 1

## Figures and Tables

**Table 1:** Summary of the main simulations performed in this study.

Simulation	Forcefield	Repeats × duration
CG-MD, M37 + PC bilayer	Martini 2.2	6 × 1 μs
CG-MD, M37 + PC/PG bilayer	Martini 2.2	20 × 5 μs
AT-MD, M37 + PC/PG bilayer^a^	GROMOS 53A6	9 × 200 ns
AT-MD, M37 in water	GROMOS 53A6	3 × 200 ns
AT-MD, M37 + tributyrin	GROMOS 54A7	3 × 100 ns
Steered AT-MD, M37 in water: CV = Distance^1^	GROMOS 54A7	5 × 10 ns
Steered AT-MD, M37 + tributyrin: CV = Distance^2^	GROMOS 54A7	3 × 15 ns

PC/PG bilayer = 20% DPPG + 80% DPPC. Different CVs were tested by steered MD simulations. The distance CV is defined as the distance between the lid helix and the active site flap of M37 in water.

Distance^1^ = A spring force constant of 500 kJ mol^− 1^ was used and no position restraints were applied to the protein atoms. Additional CV's were tested and are reported in the S1 text and Figs. S7 and S8.

Distance^2^ = The distance between the C4 atom of a tributyrin molecule and the Oγ atom of the catalytic Ser174 was used to study substrate binding in M37 (Fig. 7), performing SMD simulations with a spring force constant of 2000 kJ mol^− 1^nm^− 2^.

a Three replicates of each binding orientation of M37 in the bilayer (*Up*, *Angled* and *Down*) were simulated, totalling nine individual simulations of 200 ns.

## Appendix A Supplementary data

Supplementary material Image 2
