# Supplementary material for: Interfacial activation of M37 lipase: A multi-scale simulation study
Source: Biochim Biophys Acta. 2017 Mar;1859(3):340–9. doi: 10.1016/j.bbamem.2016.12.012 (PMC5287222; doi:10.1016/j.bbamem.2016.12.012)
Supplement: Supplementary file 2 — Supplementary material [file mmc2.pdf]

Supporting information for:

INTERFACIAL ACTIVATION OF M37 LIPASE: A MULTI-SCALE SIMULATION STUDY

Nathalie Willems[1], Mickaël Lelimousin[1,2], Heidi Koldsø[1,3], Mark S.P. Sansom[1]*

Elastic network parameterisation

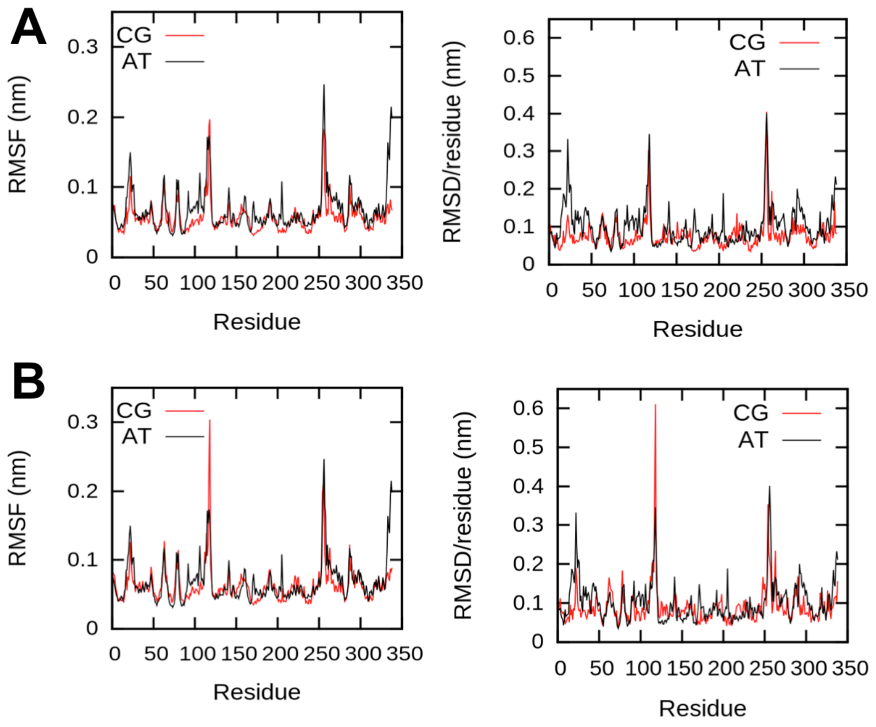

Fig S1: RMSF and RMSD values calculated for Cα particles of M37 in water for the AT-MD and CG-MD simulations. The following ENM parameters for the CG simulations were tested: A) 550 kJ mol^{-1} force constant and a 0.95 nm cut-off radius (left), or B) a 500 kJ mol^{-1} force constant and a 0.95 nm cut-off radius.

CG M37-pure PC bilayer simulations

We performed initial test simulations of M37 positioned above either pure PC bilayers (zwitterionic) or PG-containing bilayers (anionic). Simulations of M37 with pure PC bilayers did not result in sustained interactions between the protein and the membrane (Fig S2). Further test simulations were performed for a different starting orientation of M37 positioned above the pure PC bilayers, exhibiting very similar results. No binding events were seen from the total 6 replicate ensemble with pure PC bilayers. We therefore only report simulations of M37 with PG-containing bilayers, and refer only to these in the below supporting information.

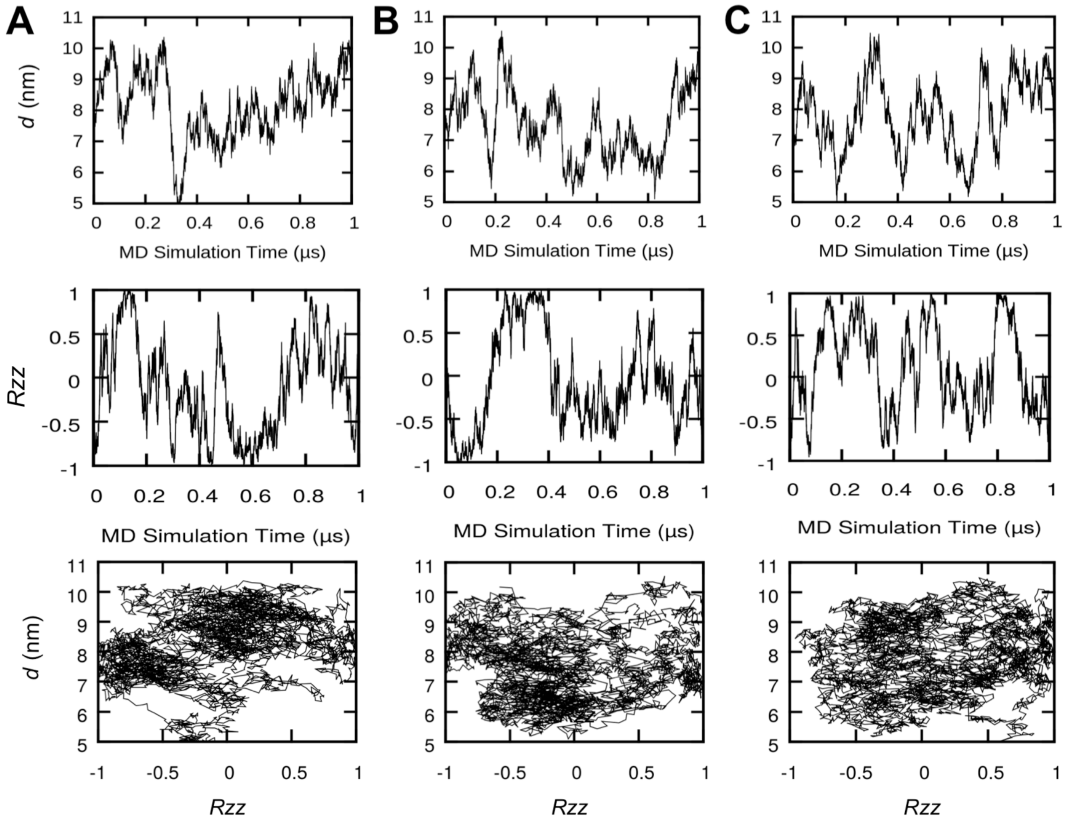

Fig S2: Interaction analysis of three replicate CG-MD simulations (A-C) of M37 with pure PC bilayers. *Top*: Time evolution of the centre of mass (COM) distance between the protein and the bilayer. *Middle*: Time evolution of the rotation matrix calculated for the protein with respect to a reference orientation (*Up*, see main manuscript). *Bottom*: COM distance between the protein and the bilayer plotted against the same rotation matrix calculation shown in the middle row. A further three replicate simulations were performed in a different starting orientation, exhibiting very similar results. None of the PC bilayer simulations resulted in sustained binding events.

CG M37-bilayer simulations: distance analysis

20 CG-MD simulations of M37 positioned above the anionic lipid bilayer were performed to investigate lipase binding behaviour with this interface. All 20 simulations resulted in lipase interactions with the PG-containing membrane. The time evolution of the centre of mass (COM) distance between the protein and the membrane (z-component) is reported for the ensemble in Fig. S3.

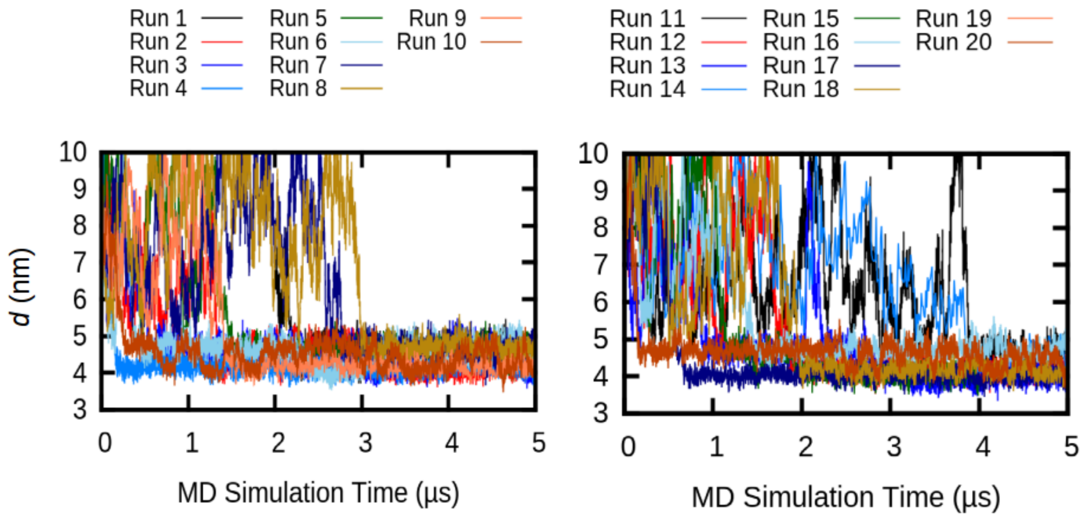

Fig. S3: Distance analysis for a 20 replicate ensemble of CG-MD simulations of M37-bilayer interactions. *Left*: Time evolution of the z-component of the COM distance between M37 and the bilayer for the first 10 replicate simulations. *Right*: Time evolution of the z-component of the COM distance between M37 and the bilayer for the last 10 replicate simulations.

CG M37-bilayer simulations: rotation matrix analysis

The rotation matrix and the COM distance between the protein and the bilayer were calculated for each of the CG M37-bilayer simulations. These data were used to generate a 2-dimensional landscape showing the normalised density of the binding orientations within the simulation ensemble (Fig. 2C). Fig. S4 shows the data used to generate Fig. 2B in the main manuscript.

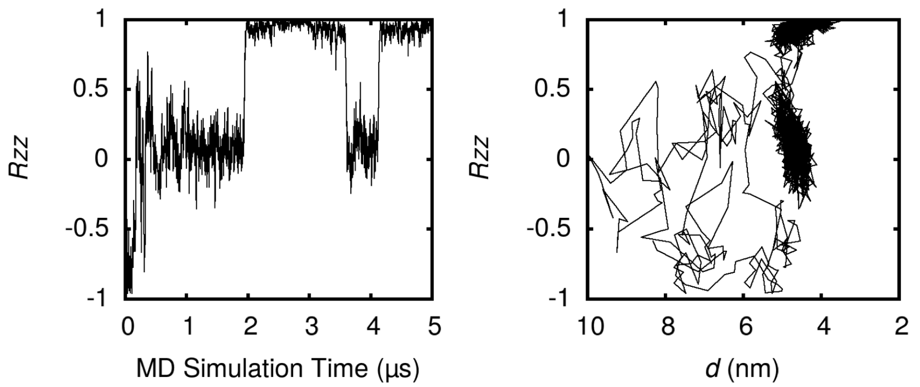

Fig. S4: Rotation matrix and distance analysis for a CG-MD simulation of M37 with anionic bilayers. *Left*: Time evolution of the *Rzz* component of the rotation matrix calculated using the analysis method shown in Fig. 2A. *Right*: The same *Rzz* component plotted against the z-component of the distance between the COM of the protein and the bilayer, indicating association of M37 with the anionic lipid bilayer.

M37-bilayer simulations: CG and AT contact analysis

To facilitate direct comparisons between the CG and AT simulations of M37 with anionic bilayers, we calculated contact maps that display which residues mediated interactions with the PC and PG lipids of the anionic bilayers (Fig. S5).

This contact analysis revealed that similar residues could be identified when comparing the CG and AT models. The AT simulations however revealed that a larger number of basic residues were involved in mediating contacts with PG lipids, particularly for the *Angled* orientation, compared to the CG simulations. Here, residues such as Lys110, Lys192, and Lys277 made contacts with PG lipids that were not identified from the CG simulations. Additionally, more residues with polar side chains were identified in contact with PC lipids for the *Angled* orientation, not observed in the CG simulations. These differences may reflect the ability of AT simulations to distinguish more subtle interactions, such as polar interactions between the choline and phosphate groups of the PC lipids with the polar side chains of Thr59, Thr139, and Thr24 (Fig. S5, *Angled* orientation). Overall however, there was good agreement between the interacting residues identified from the CG and AT simulations, particularly for contacts with PG lipids in the *Down* orientation.

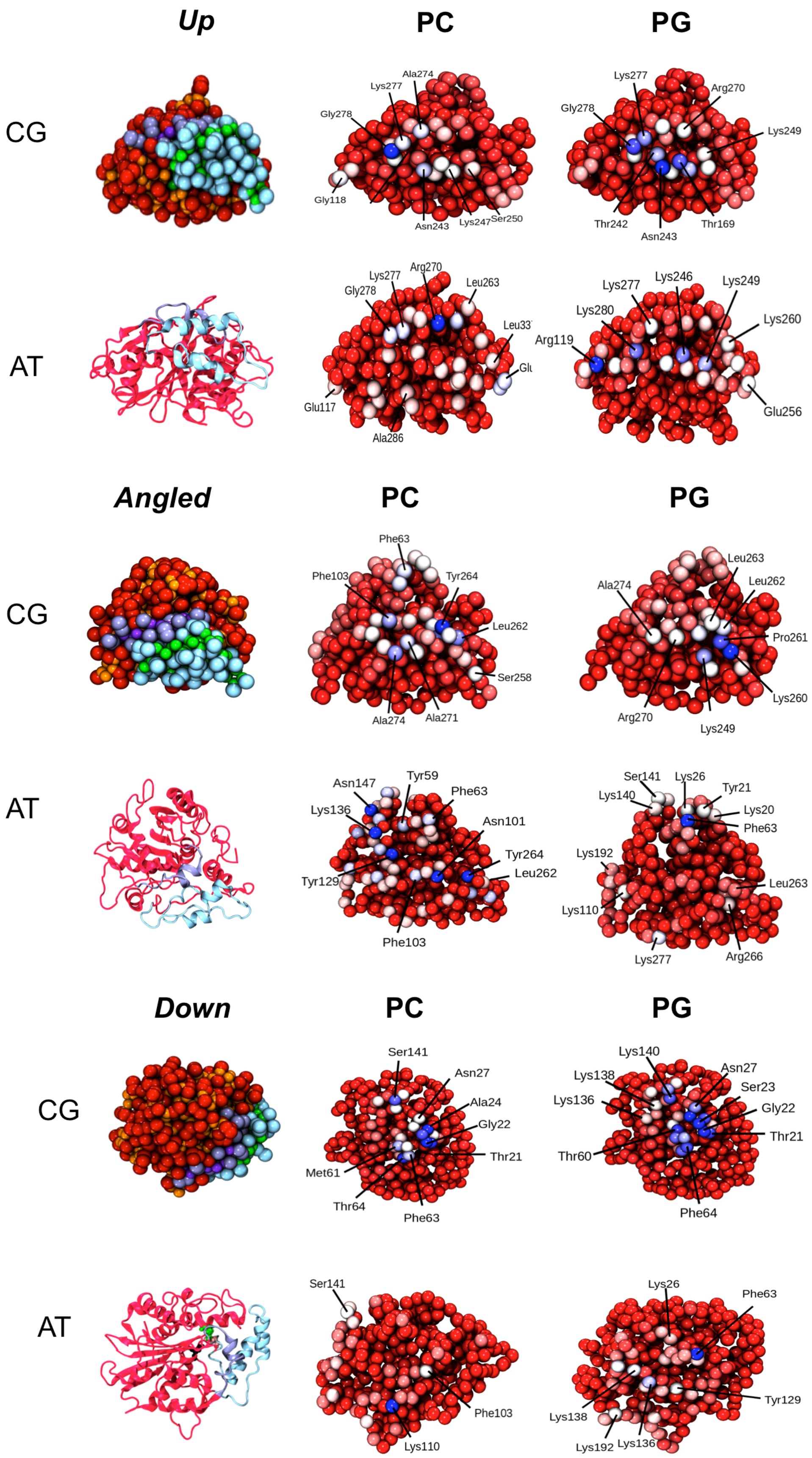

Fig. S5: Protein-lipid contacts calculated for the *Up*, *Angled,* and *Down* binding orientations observed in CG and AT M37-anionic bilayer simulations. Contacts (PC *vs* PG lipids) are shown on van der Waals representations of M37 using a blue/white/red colour scale (red = no contact, blue = greatest number of contacts). A cut-off radius of 0.8 nm was used for the CG simulations, and a 0.4 nm cut-off for the AT simulations. A reference structure showing the position of the lid and active site flap regions with respect to the contact residue maps is shown on the left.

AT-MD simulations of M37 with anionic lipid bilayers:

Final structures of M37 in either the *Up*, *Angled*, or *Down* orientation were taken from the last frame of AT-MD simulations of the protein with anionic lipid bilayers. These structures were aligned with the original closed crystal structure of M37 (PDB: 2ORY), indicating that neither the lid or active site flap region of the lipase were significantly displaced as a function of interfacial interactions with the bilayer (Fig. S6). The increase in RMSD in the lid region observed during simulations of M37 in the *Down* orientation and in water is therefore attributed to the partial loss of secondary structure within the α-helix of the lid region (Fig. 4B).

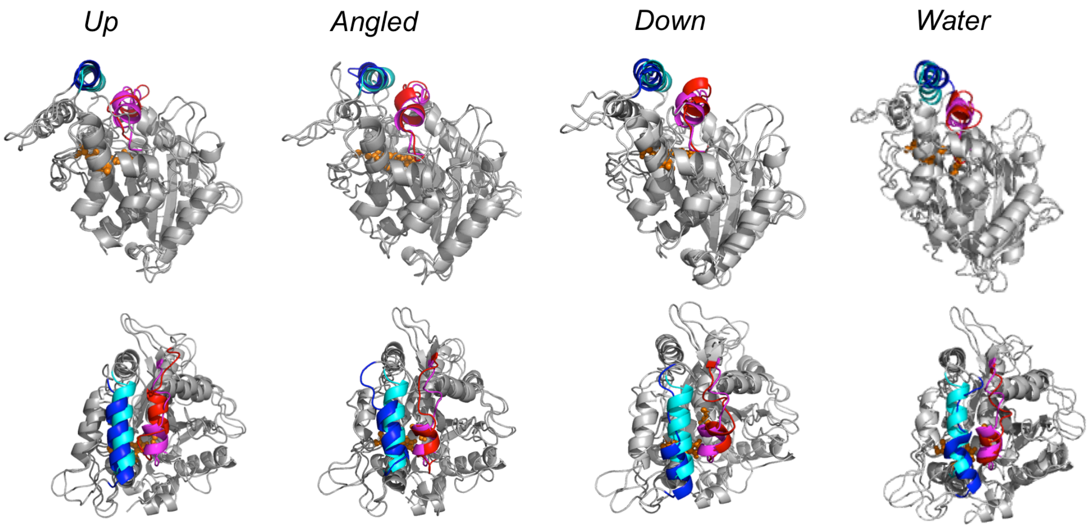

Fig. S6: Structural alignment of the final structure of M37 from atomistic simulations with anionic bilayers, and in water, with the M37 crystal structure. Alignment of the structures is shown as a side view (top panel) and top down view (bottom panel). The images represent the last frame of AT-MD simulation of M37 with anionic bilayers in different binding orientations (*Up, Angled*, and *Down*), and M37 in water, in the absence of an interface. The lipase is shown as cartoon representations (grey), the lid is coloured in blue and the active site flap in red for the simulated structures (200 ns). The same regions are shown in cyan and magenta, respectively, for the crystal structure. The catalytic triad is shown as orange van der Waals spheres.

AT SMD M37-water simulations:

Different initial CVs were tested in order to probe the functional motions of M37 (see below). It was found that a distance CV, defined as the distance between the COM of lid and active site flap helices, resulted in the most stable SMD simulations (Fig. 5).

1. Pseudo-dihedral CV

Initially, a pseudo-dihedral angle CV, defined between Cα atoms located in the hinge regions of the active site flap, was simulated (Fig. S7). The simulations employing the pseudo-dihedral angle CVs (various definitions were tested) were globally unstable and did not show any significant movements of the active site flap region (performed in water).

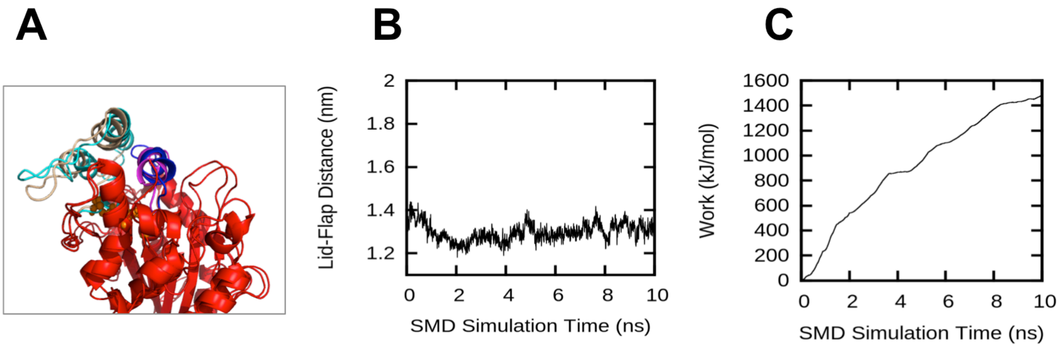

Fig. S7: Structures and analysis of a SMD simulation of M37 in water using a pseudo-dihedral angle CV. (A) Superimposed cartoon structures of M37 (red) showing the position of the lid helices and active site flap the start (cyan and magenta respectively) and end (orange and blue respectively) of the SMD simulation. **(B)** Time evolution of the COM distance between the lid helix and the active site flap helix. **(C)** Time evolution of the cumulated work performed during the SMD simulation.

2. Distance CV:

Position restrained protein: To further investigate the unexpected stability of the active site flap region of M37, SMD simulations were performed with position restraints on all protein atoms except those within the active site flap helix. Here, the CV was defined as the COM distance between the lid and active flap helices. Spring force constants (Fc) of 500 kJ mol^{-1}, 1000 kJ mol^{-1}, and 2000 kJ mol^{-1} were used to extend the distance CV, however only negligible movement of the flap helix were observed in all case (Fig. S8).

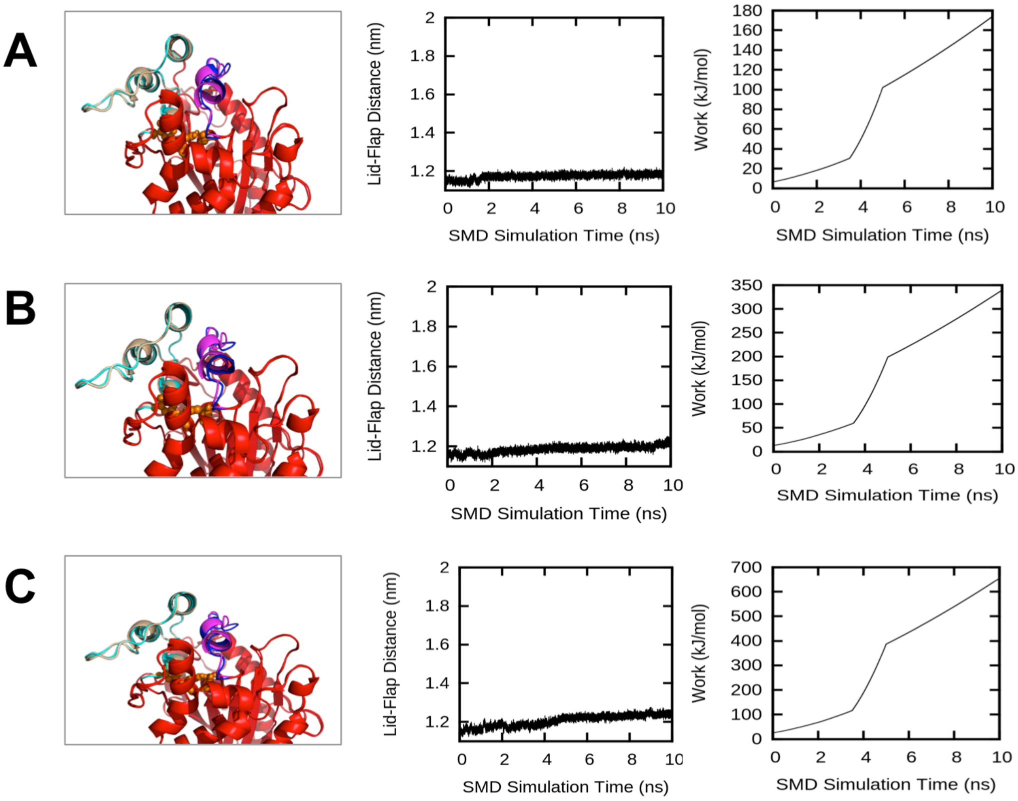

Fig. S8: SMD simulations of M37 in water using a distance CV defined between the lid helix and the active site flap helix. Three different spring force constant were tested: **(A)** 500 kJ mol^{-1}, **(B)** 1000 kJ mol^{-1} and **(C)** 2000 kJ mol^{-1}. In each simulation the protein atoms were position restrained except those of the active site flap region. *Left*: Aligned cartoon structures of M37 (red) showing the position of the lid and active site flap regions at the start (cyan and magenta respectively) and end (orange and blue respectively) of one of the SMD simulations. *Middle*: Time evolution of the COM distance between the lid helix and the active site flap helix. *Right*: Time evolution of the cumulated work performed during the SMD simulations.

No position restraints: Different Fc's were used to perform unrestrained SMD simulations using the distance CV. It was found that a Fc of 500 kJ mol^{-1} resulted in the most stable simulations. Five repeats were performed using this Fc with different initial velocities to test reproducibility. The replicate simulations showed very similar results, in particular regarding the displacement of the lid region. Fig. S9 and S10 show the time evolution of the contacts between the front helix of the lid and active site flap helix (Fig. S9), as well as the contacts between these regions at the start and end of the SMD simulations (Fig. S10). Contact analysis is shown for 3 out of the 5 replicates that were performed.

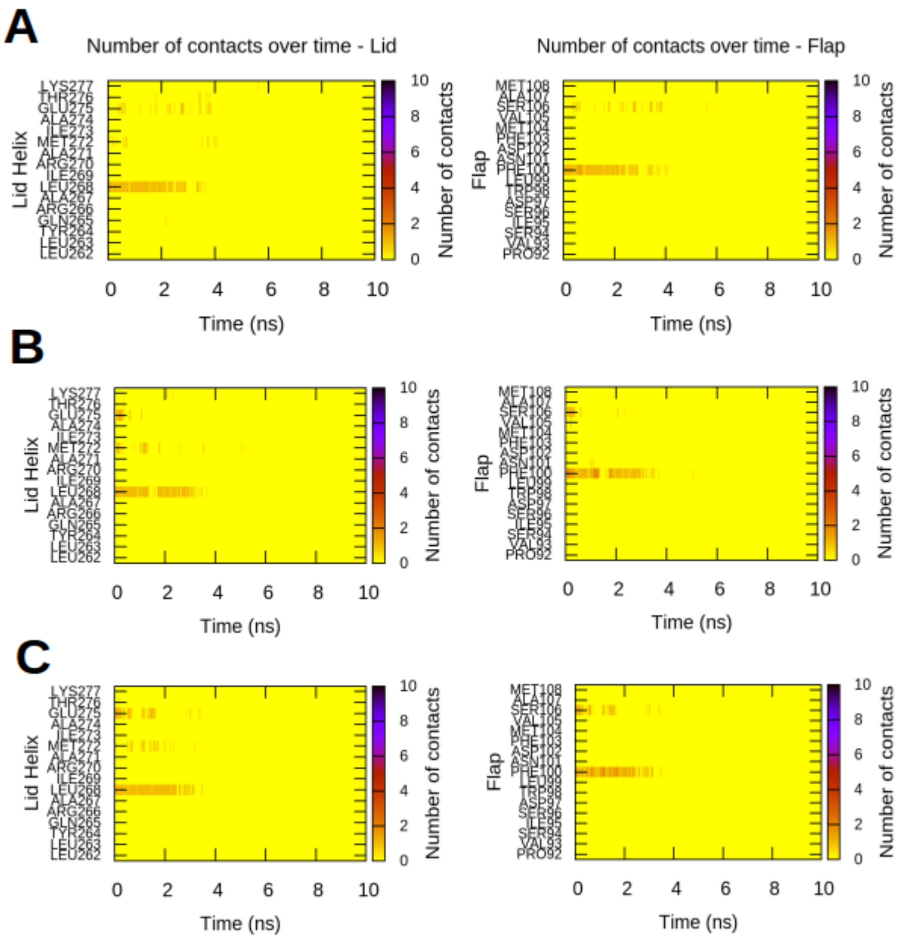

Fig. S9: Time evolution of the contacts calculated for three of five unrestrained replicate SMD simulations of M37 in water using the lid-flap distance CV. A 0.4 nm cut-off was used to calculate the contacts between residues of the lid and active site flap helices.

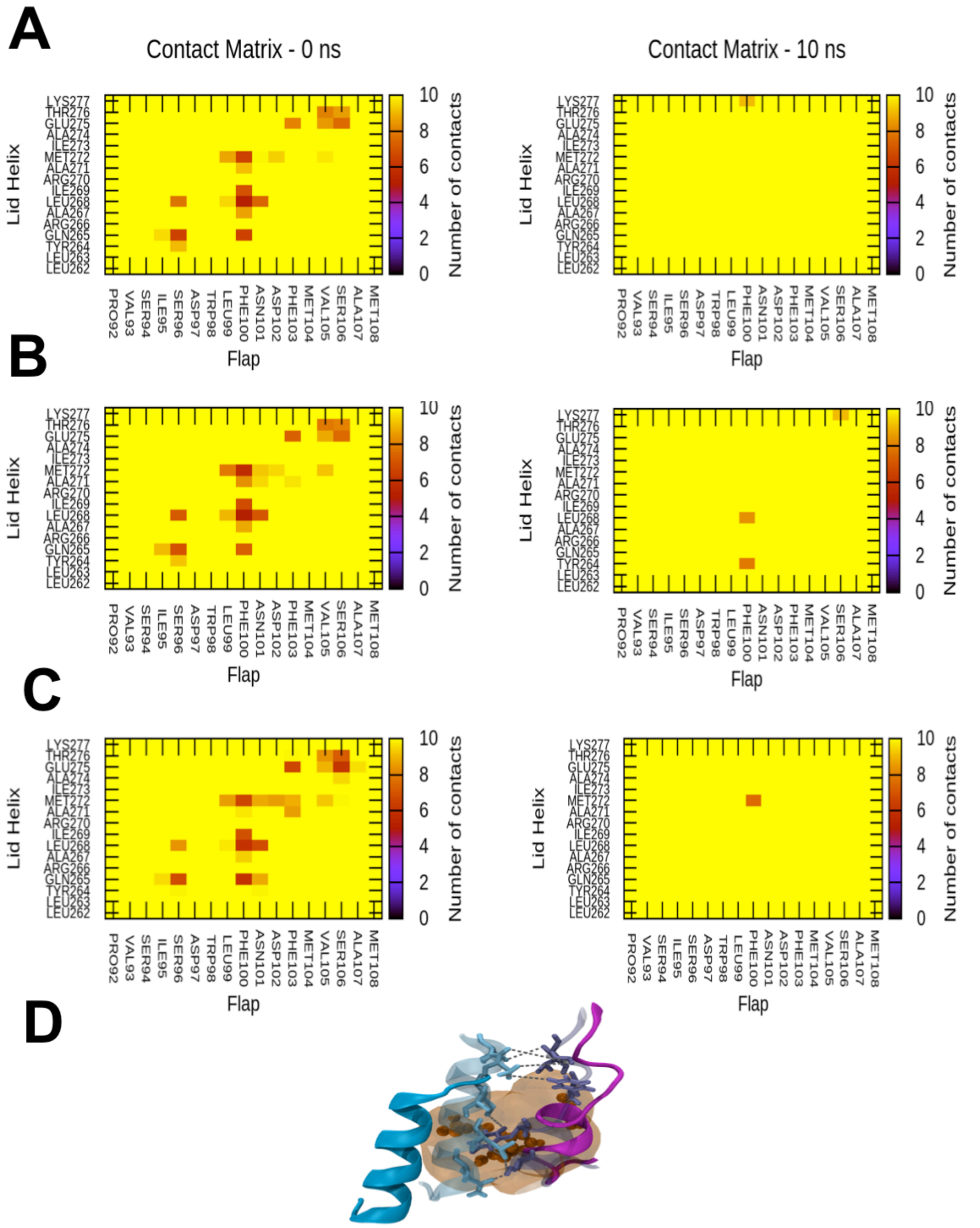

Fig. S10: Contact matrices calculated for three of five unrestrained replicate SMD simulations of M37 in water using the lid-flap distance CV (A-C). The matrices show contacts for the first and final frames of the SMD simulations. A 0.4 nm cut-off was used to calculate the contacts between residues of the lid and active site flap helices. **(D)** Position of the lid helix (cyan) and active site flap (purple) region at the start (transparent) and end (opaque) of an SMD simulation. The active site pocket is shown as an orange surface, and the underlying catalytic residues as orange van der Waals spheres. The hydrophobic residues within the helices are shown as sticks. Initial contacts are indicated by the dotted lines.

The main contacts made between the lid and the active site flap regions involved both polar and hydrophobic residues, mainly between Glu265-Ser96, Leu268/Met272-Phe100 and Glu275-Ser106. These interactions broke as the helices moved away from each other, and the hydrophobic residues became exposed, as may be expected upon lipase interaction with a hydrophobic interface resulting in lipase activation.

Docking studies

Docking calculations were performed to identify possible binding positions of a tributyrin substrate molecule, using the opened structure of M37 obtained at the end of a tributyrin interface simulation. For comparison, docking was also performed on the closed form of the enzyme (crystal structure). The pose of the tributyrin substrate with the best docking score for the opened structure of M37 is shown in Fig. S11. In contrast no docking poses could be obtained for the closed state of M37 (equilibrated crystal structure).

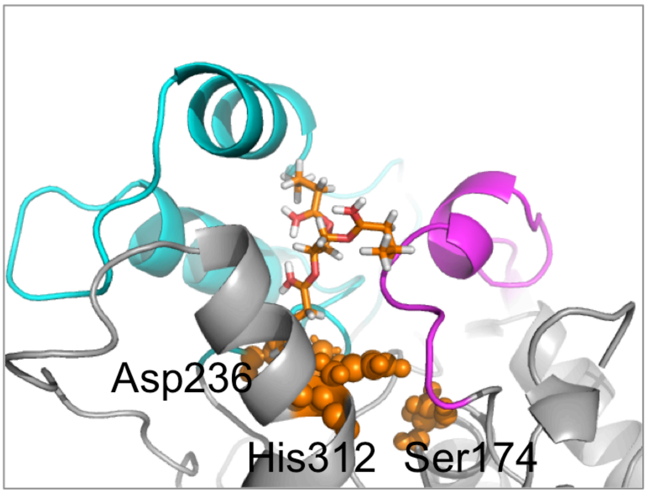

Fig. S11: The highest ranking docking pose calculated for a single tributyrin molecule within the opened structure of M37. Tributyrin is shown as orange, red, and grey sticks. The lid region is coloured in cyan and the active site flap region in magenta. The catalytic triad is shown as orange van der Waals spheres.
